# Collagenous gastritis in pediatric patients with severe follicular pangastritis and iron deficiency anemia

**DOI:** 10.1002/jpr3.70279

**Published:** 2026-10-28

**Authors:** María Juliana Rusinque Del Busto, Karina Lozano Rivera, Maira Alejandra Forero Rivera, Alejandra Calderón, Santiago Walker, Angie Vergara Espitia, José Fernando Vera Chamorro

**Affiliations:** ^1^ Universidad de los Andes, Fundación Santa Fe de Bogotá Grupo de investigación PediaFe Bogotá Colombia; ^2^ Gastroenterología Pediátrica, Fundación Santa Fe de Bogotá Bogotá Colombia; ^3^ Gastroenterología Pediátrica Universidad El Bosque Bogotá Colombia; ^4^ Patología Universidad de los Andes Bogotá Colombia; ^5^ Oncohematología Pediátrica, Fundación Santa Fe de Bogotá Bogotá Colombia; ^6^ Patología Gastrointestinal, Fundación Santa Fe de Bogotá Bogotá Colombia

**Keywords:** abdominal pain, gastric mucosa, nodular gastritis, refractory iron deficiency, subepithelial collagen deposition

## Abstract

Collagenous gastritis is a rare disorder characterized by subepithelial collagen deposition in the gastric mucosa and is frequently associated with iron deficiency anemia. We report two girls aged 8 and 16 years with persistent iron deficiency anemia, abdominal pain, bloating, and constipation. Upper endoscopy revealed severe follicular pangastritis in both patients. Histopathology demonstrated collagenous gastritis, with subepithelial collagen bands measuring 23 and 18 μm, respectively, accompanied by eosinophilic infiltration and absence of *Helicobacter pylori* infection. One patient had a history of cow's milk allergy and autoimmune family history, whereas the second had positive celiac serology with negative HLA‐DQ2/DQ8. Individualized management included dietary modification, iron replacement, proton pump inhibitor therapy, systemic corticosteroids in one patient, and oral budesonide in the other. Both patients showed clinical improvement and recovery from iron deficiency anemia. These cases highlight collagenous gastritis as an important diagnostic consideration in children and adolescents with refractory iron deficiency anemia and follicular pangastritis.

## Abstract (highlights)

**What is Known**

- Collagenous gastritis is a rare disease with fewer than 400 reported cases globally.
- In children, it typically presents with iron deficiency anemia and chronic abdominal pain.
- The histological hallmark is a subepithelial collagen band thicker than 10 μm.

**What is New**

- Two pediatric cases had severe follicular pangastritis as a prominent endoscopic finding.
- Individualized treatment was associated with clinical and hematologic improvement in both patients.
- The cases add clinical and histopathological data to the limited pediatric literature on collagenous gastritis in Latin America.

## 1 INTRODUCTION

Collagenous gastritis is a rare condition characterized by the accumulation of collagen beneath the gastric epithelium. It was first described in 1989 by Coletti and Trainer.^1^ Less than 400 cases have been reported worldwide in both the pediatric population and adults; a European study estimates an approximate incidence of 0.25 per 100,000 people per year. It is considered an occasional diagnosis in pediatrics.^2^

Pediatric and adult presentations of collagenous gastritis appear to differ. Pediatric cases have more commonly been characterized by abdominal pain, iron deficiency anemia, and prominent gastric involvement, whereas adult cases have been reported with a broader spectrum of gastrointestinal manifestations with malabsorption symptoms such as diarrhea, which affects the small intestine and colon, and features colitis, with associations with autoimmune diseases.^3^

Although the etiology and pathophysiology of collagenous gastritis remain incompletely understood, it has been hypothesized that subepithelial collagen deposition may occur in response to inflammatory or immune‐mediated triggers.^4^ Associations with autoimmune conditions, including celiac disease, have also been reported.^5^

During endoscopy, no specific macroscopic sign is evident to indicate the condition, which complicates diagnosis. Confirmation relies on histopathology, revealing a subepithelial collagen band thicker than 10 μm with surface capillary entrapment.^6^ Three inflammation patterns are identified: lymphocytic, marked by more than 25 lymphocytes per 25 epithelial cells; eosinophilic, characterized by more than 30 eosinophils per high‐power field; and atrophic, characterized by moderate to severe oxyntic gland atrophy, smooth muscle infiltration into the lamina propria, pyloric metaplasia, and intestinal metaplasia.^6^, ^7^

There is currently no standardized treatment algorithm for pediatric collagenous gastritis, and management is generally individualized according to clinical severity and associated conditions. Options include dietary restrictions, proton pump inhibitors, sucralfate, iron supplements, corticosteroids, and topical or viscous budesonide. Available evidence is limited, and treatment responses have varied across reported cases.^8^, ^9^

## 2 METHODS

This study reports on two pediatric patients who presented to the Fundación Santa Fe de Bogotá. Diagnostic evaluation included laboratory tests (complete blood count, ferritin, albumin), upper gastrointestinal endoscopy, and colonoscopy. Gastric biopsies were obtained and analyzed using hematoxylin and eosin (H&E), Masson's trichrome, and reticulin stains. Subepithelial collagen thickness was measured in micrometers (μm). The patients were treated and followed for clinical and laboratory response.

### 2.1 Ethics statement

This case report was approved by the Institutional Ethics Committee of Fundación Santa Fe de Bogotá. Written informed consent for publication was obtained from the patients' parents or legal guardians. Patient anonymity and confidentiality were preserved throughout all stages of data collection, manuscript preparation, and publication, and all identifying information was removed. This guarantees, throughout all stages of its execution and publication, the anonymity and confidentiality of the patient's personal data.

### 2.2 Study dates

The data collection for these cases took place between January 2023 and December 2024.

### 2.3 Statistical analysis

Does not apply.

## 3 RESULTS

### 3.1 Case 1

An 8‐year‐old girl presented with a 7‐month history of lower abdominal pain, hyporexia, mucocutaneous pallor, abdominal distension, and constipation. She had a personal history of atopy and a family history of autoimmune diseases on her paternal side, including Sjögren syndrome and Type 1 diabetes mellitus. She had no other symptoms or signs of nutritional deficiency. Initial tests showed microcytic hypochromic anemia with an altered iron profile, indicating severe iron deficiency. Additionally, abdominal ultrasound revealed hepatosplenomegaly, while the abdominal X‐ray was normal. An endoscopic examination outside the institution identified chronic gastritis with features of collagenous gastritis, focal complete intestinal metaplasia in the antrum, and Grade 1 duodenitis with lymphoid follicle hyperplasia. Due to ongoing symptoms, a new upper endoscopy was performed (Figure 1), revealing follicular pangastritis (possible lymphocytic or autoimmune gastritis under review), follicular duodenitis, a negative rapid urease test, and a regular colonoscopy up to the cecum.

**Figure 1:**
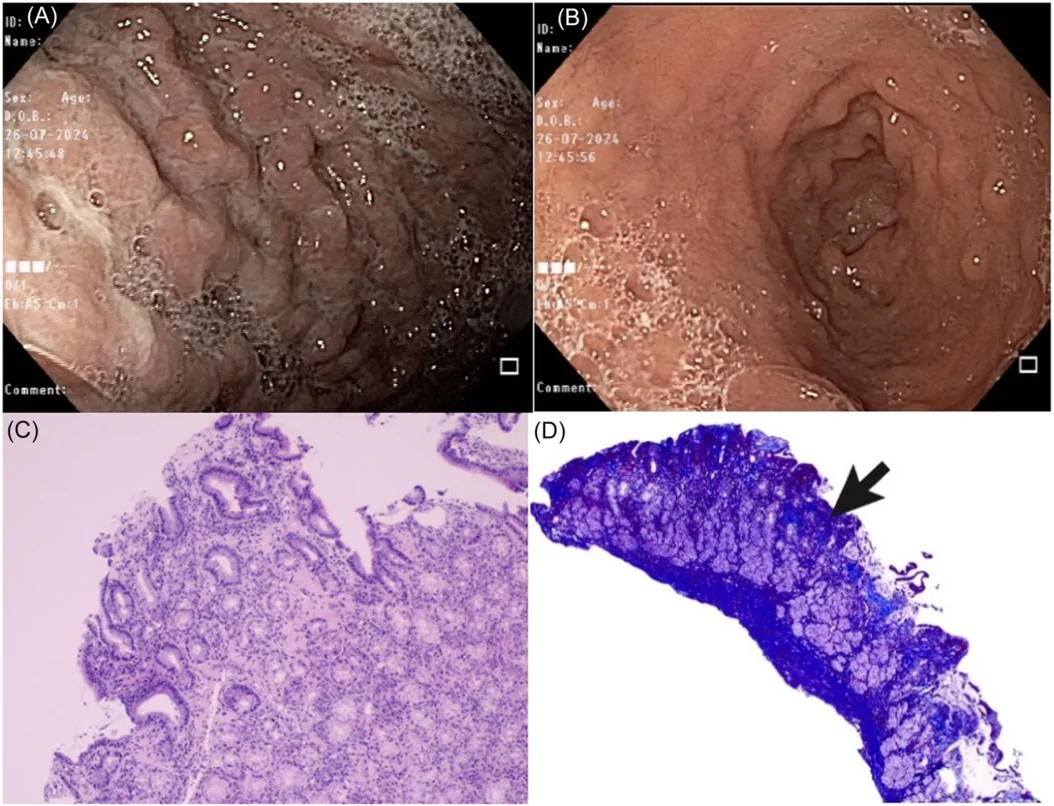
Initial esophagogastroduodenoscopy: (A) Mucosa of the body and antrum with erythematous appearance and multiple follicular lesions. (B) Mucosa of the duodenum with follicular appearance, second portion with normal appearance. (C) H&E stain at 10× magnification showing an irregular subepithelial collagen band in a background of chronic gastritis and atrophic mucosa, with splitting of the muscularis mucosa. (D) Masson's trichrome stain highlights the subepithelial collagen band, with the arrow indicating a thickness greater than 10 μm. H&E, hematoxylin and eosin.

Antral biopsies revealed segmental subepithelial thickening with collagen deposition, highlighted by Masson's trichrome stain and exceeding 10 μm in some areas, together with superficial epithelial erosion, consistent with collagenous gastritis (Figure 1). The lamina propria was expanded by a lymphoplasmacytic infiltrate with prominent eosinophils, corresponding to an eosinophil‐rich inflammatory pattern previously described in collagenous gastritis. In the gastric body, severe oxyntic mucosal atrophy with oxyntic cell loss, antralization of the mucosa, and linear neuroendocrine cell hyperplasia was observed. These findings were compatible with an atrophic pattern of collagenous gastritis, which may histologically resemble autoimmune gastritis. The presence of subepithelial collagen deposition and the absence of serologic evidence of autoimmune gastritis supported collagenous gastritis as the final diagnosis.

During hospitalization, parenteral iron supplementation was required because of severe iron deficiency anemia. Further evaluation, including hemoglobin electrophoresis and an osmotic fragility test, showed no evidence of an alternative hematologic disorder. An autoimmune workup was negative, including parietal cell and intrinsic factor antibodies.

Following confirmation of the diagnosis, cow's milk protein was eliminated from the diet, an elemental formula was initiated, and treatment with systemic corticosteroids and a proton pump inhibitor was started concurrently because of the severity of her symptoms, mucosal inflammation, and iron deficiency anemia. After 10 days of hospitalization, the patient was discharged; systemic corticosteroids were subsequently tapered, while dietary modification and proton pump inhibitor therapy were continued, and has been under outpatient follow‐up for about a year with a pediatric gastroenterologist, showing significant improvement.

### 3.2 Case 2

A 16‐year‐old female patient with no significant personal or family history presented with a 2‐year history of abdominal pain, constipation with intermittent episodes of loose, explosive stools, and abdominal distension. Laboratory evaluation revealed iron deficiency anemia refractory to oral iron supplementation. Given the change in bowel habits and persistent gastrointestinal symptoms, further evaluation was performed. Laboratory studies showed elevated gliadin Immunoglobulin G (IgG) (73.3 U/mL), tissue transglutaminase Immunoglobulin A (IgA) antibodies (29 U/mL), and fecal calprotectin (798.4 μg/g). Anti‐neutrophil cytoplasmic antibodies, endomysial IgG antibodies, anti‐parietal cell antibodies, and anti‐intrinsic factor antibodies were negative. Gastrin was 92.5 pg/mL.

Given the positive celiac serology and the clinical suspicion of celiac disease, a gluten‐free diet was initiated; HLA‐DQ2/DQ8 testing was subsequently negative, making celiac disease unlikely, so dietary restrictions were lifted. During this period, treatment with a proton pump inhibitor, oral iron supplementation, and probiotics was initiated. Hematology follow‐up was continued because of persistent iron deficiency anemia, and vitamin D supplementation was added.

Because of the persistent symptoms and refractory iron deficiency anemia, an upper gastrointestinal endoscopy was subsequently performed. It revealed severe follicular pangastritis (Figure 2) without evidence of *Helicobacter pylori* infection. Histopathological examination demonstrated a moderate predominantly mononuclear inflammatory infiltrate in the lamina propria of the gastric body, with focal activity and a thick subepithelial collagen band associated with denuded superficial epithelium (Figure 2). Masson's trichrome staining confirmed subepithelial collagen deposition, establishing the diagnosis of collagenous gastritis.

**Figure 2:**
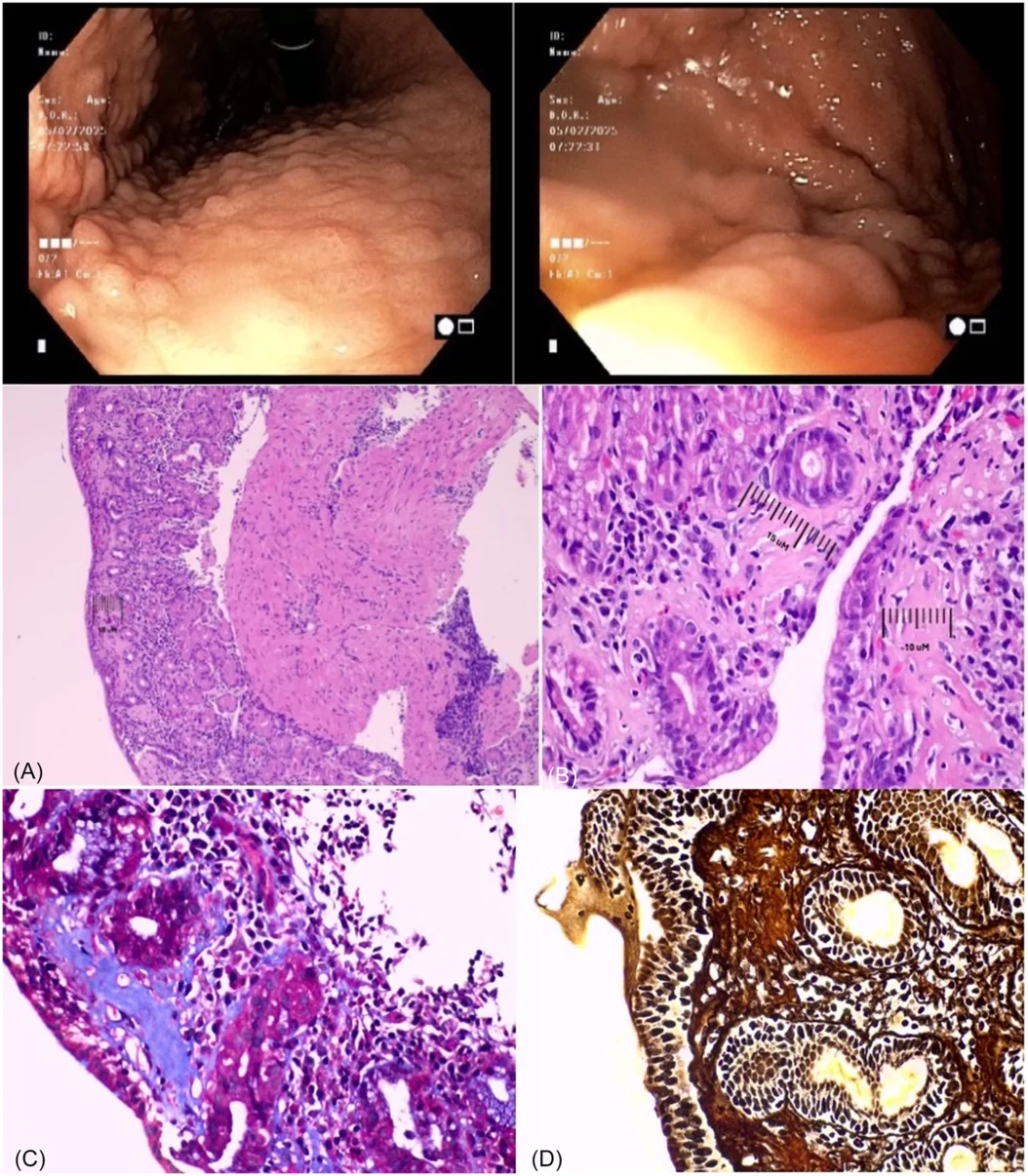
Initial esophagogastroduodenoscopy: (A) Mucosa of the body and antrum appearing erythematous with multiple edematous follicular lesions. (B) Mucosa of the duodenum appearing normal. (C) H&E stain (10×) and (D). H&E stain (40×) of antral‐corpus mucosa with collagenous pattern gastritis, showing a discontinuous subepithelial band greater than 10 microns in the setting of chronic gastritis without atrophy. (E) Masson's trichrome stain (40×) and (F). Reticulin stain (40×) reveals abundant subepithelial collagen deposition (blue and brown, respectively) with entrapment of inflammatory cells. H&E, hematoxylin and eosin.

Following the diagnosis, treatment was adjusted to include parenteral iron, oral budesonide, a proton pump inhibitor, and Vitamin D supplementation. The patient subsequently became asymptomatic, with normal menstrual cycles and progressive recovery from iron deficiency anemia, as demonstrated by the most recent complete blood count.

## 4 DISCUSSION

Collagenous gastritis is a rare gastric disorder characterized by subepithelial collagen deposition and is particularly uncommon in the pediatric population. Although the number of reported pediatric cases has increased in recent years,^2^ the largest reported cohorts are found in Sweden, Denmark, and France, with some cases also reported in the United States and the Middle East.^2^, ^6^, ^10^ The disease remains uncommon, and its pathogenesis is not completely understood. Previous pediatric reports have described a predominance among females and presentation during school age or adolescence, frequently associated with abdominal pain and iron deficiency anemia. Autoimmune conditions and immune‐mediated mechanisms have also been described in some patients.^11^, ^12^ In the present report, both patients were adolescent or school‐aged girls who presented with persistent iron deficiency anemia and gastrointestinal symptoms. These cases add to the limited pediatric literature from Latin America and highlight the potential association between collagenous gastritis and severe follicular gastric inflammation.

The clinical presentation of collagenous gastritis can be variable and nonspecific, which may contribute to diagnostic delay. Abdominal pain, iron deficiency anemia, pallor, and gastrointestinal symptoms have been frequently reported in pediatric patients.^8^, ^13^ In our patients, refractory iron deficiency anemia was a prominent feature, accompanied by abdominal pain, abdominal distension, and altered bowel habits. These findings are clinically relevant because iron deficiency anemia may initially be attributed to more common causes, particularly in children and adolescents.^13^ Persistent or unexplained anemia, especially when associated with chronic gastrointestinal symptoms and abnormal gastric mucosal findings, should therefore prompt consideration of collagenous gastritis and adequate gastric biopsies.^14^

Histopathological evaluation is essential for diagnosis because the endoscopic appearance is not specific. The defining feature is a subepithelial collagen band greater than 10 μm, which can be demonstrated using special stains such as Masson's trichrome.^6^, ^15^ Previous studies have described lymphocytic, eosinophilic, and atrophic histopathological patterns, and more than one pattern may coexist in the same patient.^6^, ^9^ This heterogeneity is important because some patterns may resemble other gastric disorders and may complicate the diagnostic process. In our first patient, the antral mucosa showed an eosinophil‐rich collagenous pattern, whereas the gastric body demonstrated severe atrophy with an autoimmune‐like pattern. These findings illustrate the histopathological heterogeneity of collagenous gastritis and emphasize the importance of correlating the microscopic findings with the clinical and endoscopic presentation. Previous reports have also suggested that histological abnormalities may persist despite clinical improvement, indicating that symptomatic response does not necessarily represent histological remission.^6^ However, because standardized recommendations for histological follow‐up are lacking, the optimal role and timing of repeat endoscopy remain uncertain.

There is currently no standardized treatment algorithm for pediatric collagenous gastritis, and management is generally individualized according to clinical severity and associated conditions.^16^, ^17^ Reported therapeutic approaches include proton pump inhibitors, iron supplementation, dietary modifications, systemic corticosteroids, and topical or viscous budesonide, with variable clinical and histological responses.^16^, ^18^ Although budesonide has shown potential benefit in some reports, current evidence is insufficient to consider it a standard therapy.^19^

The present cases highlight several clinically relevant features of pediatric collagenous gastritis. First, persistent iron deficiency anemia may be an important presenting feature and should prompt consideration of this diagnosis when common causes have been excluded. Second, severe follicular pangastritis was a prominent endoscopic finding in both patients, suggesting that this pattern may be a useful clue to underlying collagenous gastritis, although it is not specific. Finally, the coexistence of different histopathological patterns within the same patient emphasizes the importance of adequate gastric sampling and careful pathological evaluation. Given the rarity of the disease and the absence of standardized treatment or follow‐up recommendations, additional pediatric cases and longitudinal studies are needed to better define its natural history, optimal management, and relationship with immune‐mediated gastric disorders.

## 5 CONCLUSION

Collagenous gastritis should be considered in children and adolescents with persistent or refractory iron deficiency anemia accompanied by chronic gastrointestinal symptoms, particularly when upper gastrointestinal endoscopy reveals nonspecific or follicular gastric mucosal abnormalities. Because the diagnosis depends on histopathological assessment, adequate gastric biopsies are essential when endoscopy is performed in patients with compatible clinical features. Treatment should be individualized, as no standardized therapeutic algorithm currently exists. Further pediatric studies are needed to define optimal treatment and the role of long‐term clinical and histological follow‐up.

## CONFLICT OF INTEREST STATEMENT

The authors declare no conflicts of interest.

## Data Availability

The data that support the findings of this study are available on request from the corresponding author. The data are not publicly available due to privacy or ethical restrictions.
